# Prevalence and clonal relationship of ESBL-producing *Salmonella* strains from humans and poultry in northeastern Algeria

**DOI:** 10.1186/s12917-017-1050-3

**Published:** 2017-05-15

**Authors:** Samia Djeffal, Sofiane Bakour, Bakir Mamache, Rachid Elgroud, Amir Agabou, Selma Chabou, Sana Hireche, Omar Bouaziz, Kheira Rahal, Jean-Marc Rolain

**Affiliations:** 1 GSPA research Laboratory (Management of Animal Health and Productions), Institute of Veterinary Sciences, University Frères Mentouri Constantine 1, Constantine, Algeria; 2 Institute of Veterinary and Agronomic Sciences, University Chadli Bendjedid, Eltarf, Algeria; 3 Unité de recherche sur les maladies infectieuses et tropicales émergentes (URMITE), UM 63, CNRS 7278, IRD 198, INSERM 1095, IHU Méditerranée Infection, Faculté de Médecine et de Pharmacie, Aix-Marseille-Université, Marseille, France; 4 Institute of Veterinary and Agronomic Sciences, University Hadj Lakhdar, Batna, Algeria; 5 PADESCA Research Laboratory, Institute of Veterinary Sciences, University Frères Mentouri, Constantine, Algeria; 6 Pasteur Institute, Medical Bacteriology Service, Algiers, Algeria

**Keywords:** *Salmonella*, poultry, human, serotype, antimicrobial resistance, resistance genes, clonality

## Abstract

**Background:**

The aims of this study were to investigate *Salmonella* contamination in broiler chicken farms and slaughterhouses, to assess the antibiotic resistance profile in avian and human *Salmonella* isolates, and to evaluate the relationship between avian and human Extended Spectrum β-Lactamase (ESBL)-producing isolates. *Salmonella* was screened in different sample matrices collected at thirty-two chicken farms and five slaughterhouses. The human isolates were recovered from clinical specimens at the University Teaching Hospital of Constantine (UTH). All suspected colonies were confirmed by MALDI-TOF (Matrix Assisted Laser Desorption Ionization Time OF light) and serotyped. Susceptibility testing against 13 antibiotics including, amoxicillin/clavulanic acid, ticarcillin, cefoxitin, cefotaxime, aztreonam, imipenem, ertapenem, gentamicin, amikacin, ciprofloxacin, colistin, trimethoprim/sulfamethoxazole and fosfomycin, was performed using the disk diffusion method on Mueller-Hinton agar. ESBL-production was screened by the double-disk synergy test and confirmed by molecular characterization using PCR (polymerase chain reaction) amplification and sequencing of ESBL encoding genes. Clonality of the avian and human strains was performed using the Multi Locus Sequencing Typing method (MLST).

**Results:**

Forty-five isolated avian *Salmonella* strains and 37 human collected ones were studied. Five *S*. *enterica* serotypes were found in avian isolates (mainly Kentucky) and 9 from human ones (essentially Infantis). 51.11% and 26.6% of the avian isolates were resistant to ciprofloxacin and cefotaxime, respectively, whereas human isolates were less resistant to these antibiotics (13.5% to ciprofloxacin and 16.2% to cefotaxime). Eighteen (12 avian and 6 human) strains were found to produce ESBLs, which were identified as *bla*_CTX-M-1_ (*n* = 12), *bla*_CTX-M-15_ (*n* = 5) and *bla*_TEM_ group (*n* = 8). Interestingly, seven of the ESBL-producing strains (5 avian and 2 human) were of the same ST (ST15) and clustered together, suggesting a common origin.

**Conclusion:**

The results of the combined phenotypic and genotypic analysis found in this study suggest a close relationship between human and avian strains and support the hypothesis that poultry production may play a role in the spread of multidrug-resistant *Salmonella* in the human community within the study region.

**Electronic supplementary material:**

The online version of this article (doi:10.1186/s12917-017-1050-3) contains supplementary material, which is available to authorized users.

## Background

*Salmonella* infections are a major public health problem with a significant social and economic impact. Many animal species are potential reservoirs for this bacterium, especially chickens, pigeons and reptiles [1]. Humans can commonly acquire the infection through the food chain [2]. Young and immunocompromised patients are the most exposed to dangerous complications which are generally treated with fluoroquinolones and extended-spectrum cephalosporins that are largely used in veterinary medicine [3].

In Algeria, the poultry industry has grown remarkably since 1980. However, as a result of the deleterious hygienic conditions, many infectious diseases, such as Salmonellosis, were detected in broilers and constitute a risk to the human health [4]. Due to a lack of surveillance programs, information on the prevalence of *Salmonella* and other food pathogens is incomplete. For instance, a study by the Pasteur Institute of Algeria revealed that 11% of food poisoning cases were caused by *Salmonella* spp. in 2011 [5]. 47% of these cases were mainly related to the consumption of chicken meat, as reported by Mouffok et al., [5]. Eggs and ovoproducts are also among the major sources of human infections, which require a thorough assessment and control measures for *Salmonella spp.* in the poultry industry [6]. These measures are well detailed in the Official Journal of the Algerian Republic (No. 36 of June 8, 2003); but were only applied to the poultry industry facilities in the public sector as well as to private poultry production units and facilities [7].

Selective pressure due to the misuse of antibiotics in humans and domestic livestock is one of the many factors that has led to the emergence of antibiotic resistance in commensal and pathogenic bacteria; thus, multidrug-resistant (MDR) *Salmonella* have increasingly been isolated from various food products worldwide [8].

Drug resistance is growing and has affected critically important classes of antibiotics, such as the β-lactams, which are among the most significant bactericidal antibiotics used to treat bacterial infections in humans [9]. Extended spectrum β-lactamases (ESBLs) were identified following the introduction of extended-spectrum oxyimino-cephalosporins in the 1980s for the treatment of severe human infections [10]. In veterinary medicine, a variety of these drugs are currently authorized for use, resulting in the emergence of ESBL-producing Gram-negative bacteria [11, 12].

TEM, SHV and CTX-M are the most prevalent ESBL types. Over the last decade, rates of CTX-M producing bacteria have increased worldwide in comparison with TEM and SHV [13]. This situation is rendered more complicated as these enzymes confer co-resistance to other drug classes [13, 14].

In Algeria, the first ESBLs were detected in non-typhoidal *Salmonella* in 1994 from humans [15], but until now there has been no information on the magnitude of this problem in animals and humans. This situation drove us to identify the contamination status of *Salmonella* serotypes in chicken farms and slaughterhouses, to assess their sensitivity to antimicrobials and ESBL-production and, finally, to evaluate their clonality with human pathogenic strains.

## Methods

### Study area

The present study was carried out between December 2011 and May 2013. Thirty-two chicken farms and five chicken slaughterhouses located in the province of Skikda, Algeria, took part in this survey. The choice of this study area was motivated by the size of the poultry industry and the frequent occurrence of infectious gastrointestinal pathologies as reported by local veterinary practitioners [16].

Twenty-seven poultry houses had concrete walls and floors and corrugated metal sheet roofs while the remaining five houses had earth floors with walls and roofs made of straw and reeds covered with plastic foil. None of the farming sites was fenced, allowing free access for domestic and wild animals. Their rearing capacities vary from 3500 to 20,000 birds per house.

All slaughterhouses had concrete floors with earthenware walls. The slaughtering capacity ranges from 2000 to 7000 chickens per day and the broilers are brought from different poultry farms located in several neighboring provinces. The number of chickens and slaughterhouses is statistically representative of the study region and governed by the capacity of the laboratory processing the samples.

The human *Salmonella* strains were kindly provided by the University Teaching Hospital (UTH) of Constantine and included in the study.

### Study design

For technical reasons, including access to the sampling sites, a total of 1194 samples were collected during two sampling periods (between December 2011 and September 2012 and between December 2012 and May 2013). The poultry houses were visited at two periods (when the birds were aged 15–30 days and 45–60 days).

From the chicken farms, a total of 320 samples of water were taken from drinking vessels, 160 samples of feed were taken from the feeding vessels, 330 cloacal swabs were taken and 320 droppings were collected and placed in sterile containers. 64 surface wipes (25 cm × 25 cm, AES Chemunex, Combourg, France) were also obtained from a height of 30 cm from the ground over a 400 cm^2^ area of the four walls of the poultry house and placed into sterile Stomacher bags.

The five poultry slaughterhouses were visited once for sampling. Due to limited financial resources, in each poultry slaughterhouse, we pooled individual samples to minimize study costs. The samples were randomly taken from three organs from five chickens (5 g of 5 caeca, 5 g of 5 livers, 5 g of 5 neck skins), and from the environment (one sample of carcass rinsing water, one swab from a sticking knife and one wipe from the walls). The slaughtered animals were brought from several neighboring provinces.

All samples were transported to the laboratory, on ice packs within a period not exceeding two hours, to be treated on the same day or kept in the refrigerator overnight.

### *Salmonella* isolation and identification

Bacteriological analyses were performed according to the EN/ISO 6579–2002/Amd1:2007 protocol for *Salmonella* detection in food and animal feedstuffs. 25 g of samples (droppings, feed, liver, caeca, neck skin) were individually pre-enriched with 225 mL of buffered peptone water broth (PWB) (Fluka, Sigma Aldrich, France). The swabs were individually placed in 10 mL PWB, while 100 mL of drinking and carcass rinsing water was individually mixed with 100 mL of double strength PWB for pre-enrichment according to NF U 47–101 Standard (2005) [17]. All samples were incubated at 37 °C for 18–20 h. From each pre-enrichment solution, 1 mL and 0.1 mL were respectively transferred into 10 mL of enrichment Muller-Kauffmann tetrathionate/novobiocin broth (AES Chemunex Combourg, France) and 10 mL of Rappaport Vassiliadis broth (Merck Darmstadt, Germany), incubated respectively at 37 °C and 42 °C for 24 h. Both enriched samples were then streaked on XLD (Fluka analytical Steinheim, Switzerland) and Hektoen agars (Pasteur Institute of Algeria) and incubated at 37 °C for 24 h [18]. Suspected colonies were first identified with the API 20E System (bioMérieux, France), then with MALDI-TOF (Bruker Daltonics GmbH, Germany) [19]. Confirmed *Salmonella* isolates were serotyped according to Kauffmann-White-Le Minor’s scheme [20].

### Human clinical strains

We selected 37 (non-repetitive) strains recovered from clinical specimens at the UTH of Constantine over a decade (2005–2015). These were isolated from stool samples collected from different wards and among which 26 strains derived from the diarrheic stools of infants admitted to the neonatology ward. The Main characteristics of patients are shown in Additional file 1.

The strains were confirmed using MALDI-TOF mass spectrometry for prescreening *Salmonella* species and sub-species and to identify epidemiologically important serovars which were further tested with conventional serotyping method.

### Antimicrobial susceptibility testing and ESBL detection

All human and avian strains were submitted to susceptibility testing against antibiotics using the disk diffusion method on Mueller-Hinton (MH) agar, and the results were interpreted according to the European Comitee on Antimicrobial Suceptibility Testing (EUCAST) [21] (Additional file 2). Thirteen antibiotics (Bio-Rad, France) were tested: amoxicillin/clavulanic acid AMC (20/10 μg), ticarcillin TIC (75 μg), cefoxitin FOX (30 μg), cefotaxime CTX (5 μg), aztreonam ATM (30 μg), imipenem IPM (10 μg), ertapenem ETP (10 μg), gentamicin GEN (10 μg), amikacin AK (30 μg), ciprofloxacin CIP (5 μg), colistin CT (50 μg), trimethoprim/sulfamethoxazole SXT (1.25 μg/23.75 μg) and fosfomycin FF (50 μg).

Extended spectrum β-lactamase production was screened by the double-disc synergy test (DDST) [22].

### PCR detection of ESBL genes

Total nucleic acids were extracted using a BioRobot EZ1 Advanced XL instrument (QIAGEN, Hilden, Germany) according to the manufacturer’s instructions.

Detection of β -lactamase genes (including *bla*_TEM_, *bla*_SHV_, and *bla*_CTX-M_) was carried out by polymerase chain reaction (PCR) using specific primers: *bla*_CTX-M-1_ group [23], *bla*_CTX-M-9_ group [24], *bla*_TEM_ group [25] and *bla*_SHV_ [26] and the master mix QuantiTect Probe PCR Kit (QIAGEN, Hilden, Germany).

Amplification products were detected by electrophoresis using agarose gels containing SYBR safe (Invitrogen, Leek, the Netherlands), along with a DNA molecular weight marker (BenchTop pGEM®DNA Marker, Promega, Madison, Wisconsin, USA). Visualization of gels was carried out using the BenchTop pGEM®DNA Marker (Promega, Madison, Wis- consin, USA) under ultraviolet illumination.

### ESBL genes sequencing and multilocus sequence typing

ESBL-positive PCR products were purified using the NucleoFast 96 PCR plate (Machery-Nagel EURL, France) and sequenced using the BigDye terminator chemistry on an ABI3730 automated sequencer (Applied Biosystems, Foster City, California, USA). The obtained sequences were analyzed with the ARG-ANNOT database [27].

Multilocus sequence typing (MLST) is useful in assessing the role of specific STs in human and animal disease and assessing overlap between these hosts. It was carried out by PCR amplification and sequencing of seven housekeeping genes: *thrA* (aspartokinase + homoserine dehydrogenase), *purE* (phosphoribosylaminoimidazole carboxylase), *sucA* (alpha ketoglutarate dehydrogenase), *hisD* (histidinol dehydrogenase), *aroC* (chorismate synthase), *hemD* (uroporphyrinogen III cosynthase), and *dnaN* (DNA polymerase III beta subunit), as described by Kidgell et al. 2002 [28].

Briefly, template DNA prepared from bacterial isolates was amplified by PCR with the use of an oligonucleotide sequence for seven housekeeping genes (available in the MLST database: http://mlst.warwick.ac.uk/mlst/dbs/Senterica). Sequencing with the same automated sequencer of the PCR product was carried out by the dideoxynucleotide chain termination method using the master mix QuantiTect Probe PCR Kit (QIAGEN, Hilden, Germany). Forward and reverse DNA sequences were assembled, trimmed, edited, and analyzed for each gene fragment using the ARG-ANNOT database [27]. Allelic profile and sequence type determinations were assigned according to the *Salmonella* MLST database: http://mlst.warwick.ac.uk/mlst/dbs/Senterica.

### Clonality analysis

Protein mass profiles were obtained using a Microflex LT MALDI-TOF mass spectrometer (Bruker Daltonics, Germany), with Flex Control software (Bruker Daltonics). The spectrum profiles obtained were visualized with Flex analysis v.3.3 software and exported to ClinProTools software v.2.2 and MALDI-Biotyper v.3.0 (Bruker Daltonics, Germany) for data processing (smoothing, baseline subtraction and spectra selection) and evaluation with cluster analysis.

The phyloproteomic analysis of ESBL-positive *Salmonella* strains from human and poultry origins was assessed through construction and comparison of their characteristic reference spectra (main spectra) with the MALDI-Biotyper v.3.0 software (Bruker Daltonics, Germany). Cluster analysis was performed based on pairwise comparisons of specific main spectra (MSP: mean spectra projection dendrogram) of the different strains to generate a dendrogram of similarities among spectra profiles using the software default correlation function. A distance level of 560 was selected for clustering evaluation of the isolates.

### Statistical analysis

Differences in contamination levels of poultry houses at two sampling periods (15–30 days versus 45–60 days), and the antimicrobial resistance patterns between avian and human *Salmonella* strains, were assessed by the Chi square test (at 95% CI and *p* < 0.05) or Fisher’s exact test if N is less than 20 and one expected cell is less than or equal 5. All statistical analyses were performed using IBM SPSS Statistics version 24 software (2016).

## Results

### Frequency of isolation of *Salmonella* serotypes in broilers, slaughterhouses and human samples

Forty-five *Salmonella enterica* from slaughterhouses and poultry farms and 37 of human clinical origin were studied. 34.37% of the poultry farms and all slaughterhouses were contaminated with *Salmonella* and the isolation rate varied depending on the sampling matrix. The samples taken at the age of 15–30 days were more contaminated than those collected at 45–60 days; however, the difference was not significant *(p > 0.05)*.

The isolated *Salmonella* strains belonged mainly to two serotypes: Kentucky and Heidelberg, and the remaining strains were Enteritidis, Virginia and Newport. There was an evident heterogeneous distribution of serotypes in poultry farms and slaughterhouses (Table 1).

**Table 1:** Frequency of isolation of *Salmonella enterica* subsp. *enterica* serotypes in different sample matrices from poultry farms and slaughterhouses

Serotype	Poultry farms and slaughterhouses		Samples	
	N° of positive (ID n°)	(%)	N° of positive samples	(%)
Kentucky	7 Poultry farms (F1, F6, F13, F14, F18, F20, F32)	21.87	5 cloacal swabs 6 droppings 2 wipes 3 water samples	1.51 1.87 3.12 0.93
	4 Slaughterhouses (S1, S2, S3, S4)	80	1 sticking knife 2 caeca 1 liver 1 wipe	20 8.0 4.0 20
Heidelberg	4 Poultry farms (F12, F13, F15, F27)	12.5	6 cloacal swabs 4 droppings 2 water samples 1 wipe	1.81 1.25 0.62 1.56
	(0) Slaughterhouse	0	-	-
Virginia	1 Poultry farm (F31)	3.12	1 water sample	0.31
	2 Slaughterhouses (S1,S4)	40	1 wipe 2 neck skins 1caeca	20 40 4.0
Enteritidis	3 Poultry farms (F18, F20, F27)	9.37	2 cloacal swabs 1 wipe	0.61 1.56
	1 Slaughterhouse (S3)	20	1 rinse water sample	
Newport	1 Poultry farm (F15)	3.12	1 wipe	1.56
	(0) Slaughterhouse	0	-	-
Total	11 Poultry farm (F1, F6, F12, F13, F14, F15, F18, F20, F27, F31, F32)	34.7	13 cloacal swabs 10 droppings 5 wipes 6 water samples	3.93 3.12 7.81 1.87
	5 Slaughterhouse (S1, S2, S3, S4, S5)	100	1 sticking knife 2 neck skins 1 rinse water sample 3 caeca 1 liver 2 wipe	20 8.0 4.0 12 4.0 40

*F* Farm, *S* Slaughterhouse

Among the human *Salmonella* strains, Infantis was the most frequent serotype, followed by Senftenberg, Enteritidis, Kedougou, Tyhimurium, Heidelberg, Kentucky, Ohio and Arizona. Most of these strains were from infants and the others were from adult diarrheic stools (especially *Salmonella enterica* serotypes Enteritidis and Typhimurium).

### Drug resistance patterns of the isolated *Salmonella* strains

A high frequency of resistance to ciprofloxacin (51.1%) was noted in *Salmonella* isolates from both chicken farms and slaughterhouses. These strains were resistant to cephalosporins (26.6% to cefotaxime), aztreonam (26.6%), ticarcillin (46.6%) and gentamicin (22.2%). ESBL-production was found in 26.6% of these avian isolates (11 *Salmonella* ser. Heidelberg and one *Salmonella* ser. Newport).

**Fig. 1:**
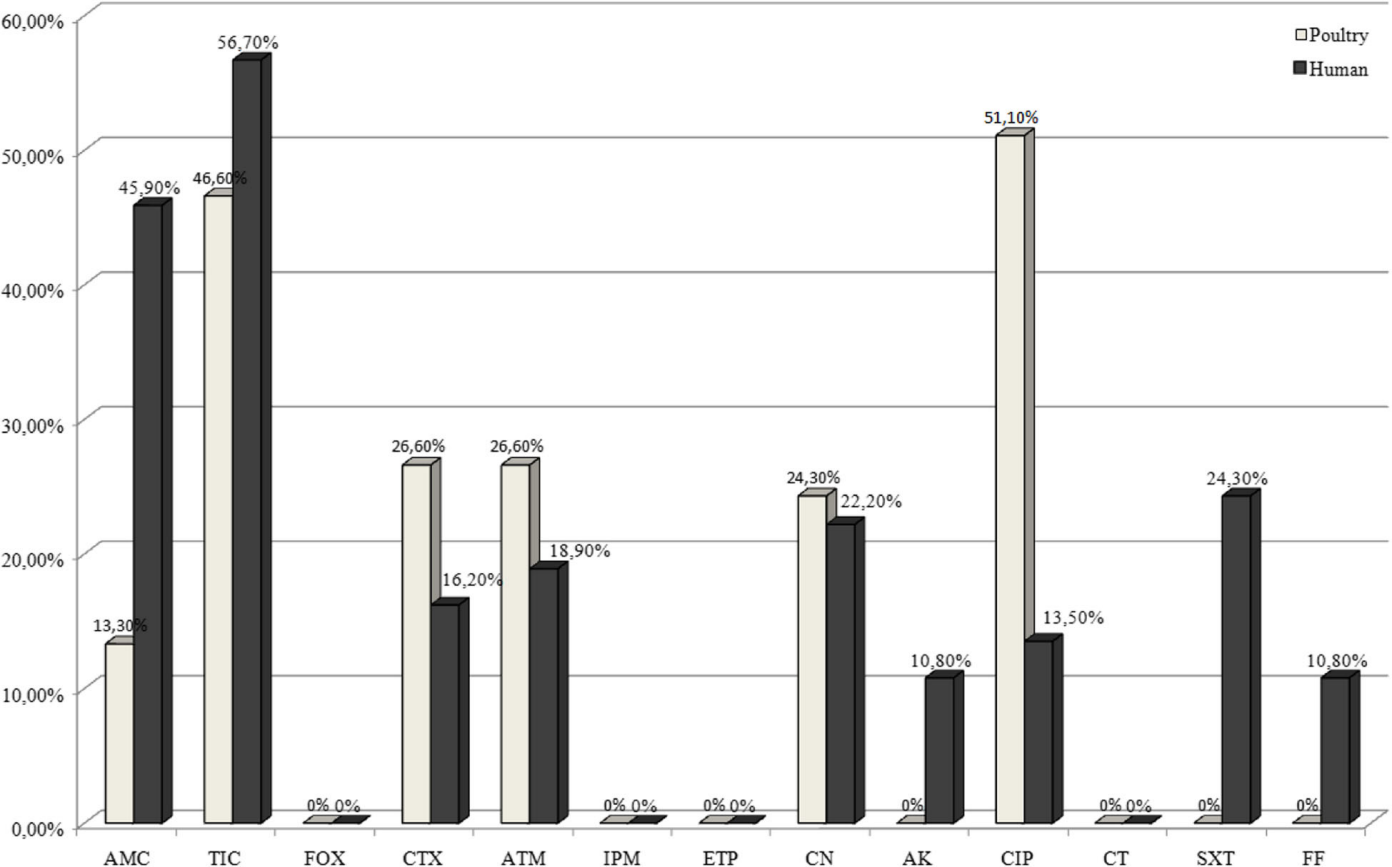
Distribution of resistance to antibiotics among *Salmonella* isolates from poultry and humans

The human *Salmonella* isolates were highly resistant to ticarcillin (56.75%), amoxicillin (45.94%) and a less extent to trimethoprim/sulfamethoxazole (24.3%), gentamicin (24.3%), aztreonam (18.9%), cefotaxime (16.2%), ciprofloxacin (13.5%) and amikacin (10.8%). Six ESBL-positive strains were detected (Fig. 1).

The difference in resistance to antibiotics, between poultry strains and human ones was significant for: amoxicillin/clavulanic acid (*P < 0.05*), gentamicin (*P < 0.05*), ciprofloxacin (*P < 0.05*) and fosfomycin (*P < 0.05*). However, it was not significant for cefotaxime, ticarcillin, aztreonam and amikacin *(P > 0.05)*.

The characteristics of the 18 ESBL-producing strains isolated from chicken farms/slaughterhouses and patients are shown in Table 2. Eleven of the *Salmonella* ser. Heidelberg strains harbored *bla*_CTX-M-1_ genes. The *bla*_TEM_ group was identified in one avian *Salmonella* ser. Newport isolate as well as in S. Heidelberg, in which it was coupled with the *bla*_CTX-M-1_ gene. Human strains harbored the *bla*_CTX-M-15_ gene in association with *bla*_TEM,_ except one strain which carried instead the *bla*_CTX-M-1_. None of the strains were positive for *bla*_CTX-M-9_ and *bla*_SHV_ genes.

**Table 2:** Antimicrobial resistance and resistant genes profiles of ESBLs producing *Salmonella enterica* strains isolated from poultry and humans

Strain ID N°	Origin	Antimicrobial Resistance Pattern	Serotype	B-Lactamase	ST
162	Poultry	TIC, CTX, ATM,	*Heidelberg*	CTX-M-1	15
163	Poultry	TIC, CTX, ATM,	*Heidelberg*	CTX-M-1	15
164	Poultry	TIC, CTX, ATM,	*Heidelberg*	CTX-M-1	15
165	Poultry	TIC, CTX, ATM,	*Heidelberg*	CTX-M-1	15
167	Poultry	TIC, CTX, ATM,	*Heidelberg*	CTX-M-1	15
169	Poultry	TIC, CTX, ATM,	*Newport*	TEM	198
170	Poultry	TIC, CTX, ATM,	*Heidelberg*	CTX-M-1	15
171	Poultry	TIC, CTX, ATM,	*Heidelberg*	CTX-M-1	15
172	Poultry	TIC, CTX, ATM,	*Heidelberg*	CTX-M-1, TEM	15
174	Poultry	TIC, CTX, ATM,	*Heidelberg*	CTX-M-1	15
177	Poultry	TIC, CTX, ATM,	*Heidelberg*	CTX-M-1	15
178	Poultry	TIC, CTX, ATM,	*Heidelberg*	CTX-M-1	15
305	Human	AMC,TIC,CTX, ATM, GEN,FF	*Senftenberg*	CTX-M-15	14
476	Human	AMC, TIC, CTX, ATM,GEN,AK,SXT	*Infantis*	CTX-M-15, TEM	38
883	Human	CTX, ATM, GEN, AK, SXT	*Heidelberg*	CTX-M-15, TEM	15
884	Human	AMC,TIC,CTX, ATM, GEN, AK, SXT	*Heidelberg*	CTX-M-15, TEM	15
YFA	Human	AMC, TIC, CTX,GEN	*Infantis*	CTX-M-1	32
1577	Human	AMC,TIC,CTX, GEN, AK, SXT	*Infantis*	CTX-M-15, TEM	16

*AMC* Amoxicillin/Clavulanic Acid, *TIC* Ticarcillin, *FOX* Cefoxitin, *CTX* Cefotaxime, *ATM* Aztreonam, *IPM* Imipenem, *ETP* Ertapenem, *GEN* Gentamicin, *AK* Amikacin, *CIP* Ciprofloxacin, *CT* Colistin, *SXT* Trimethoprim/Sulfamethoxazole, *FF* Fosfomycin

**Fig. 2:**
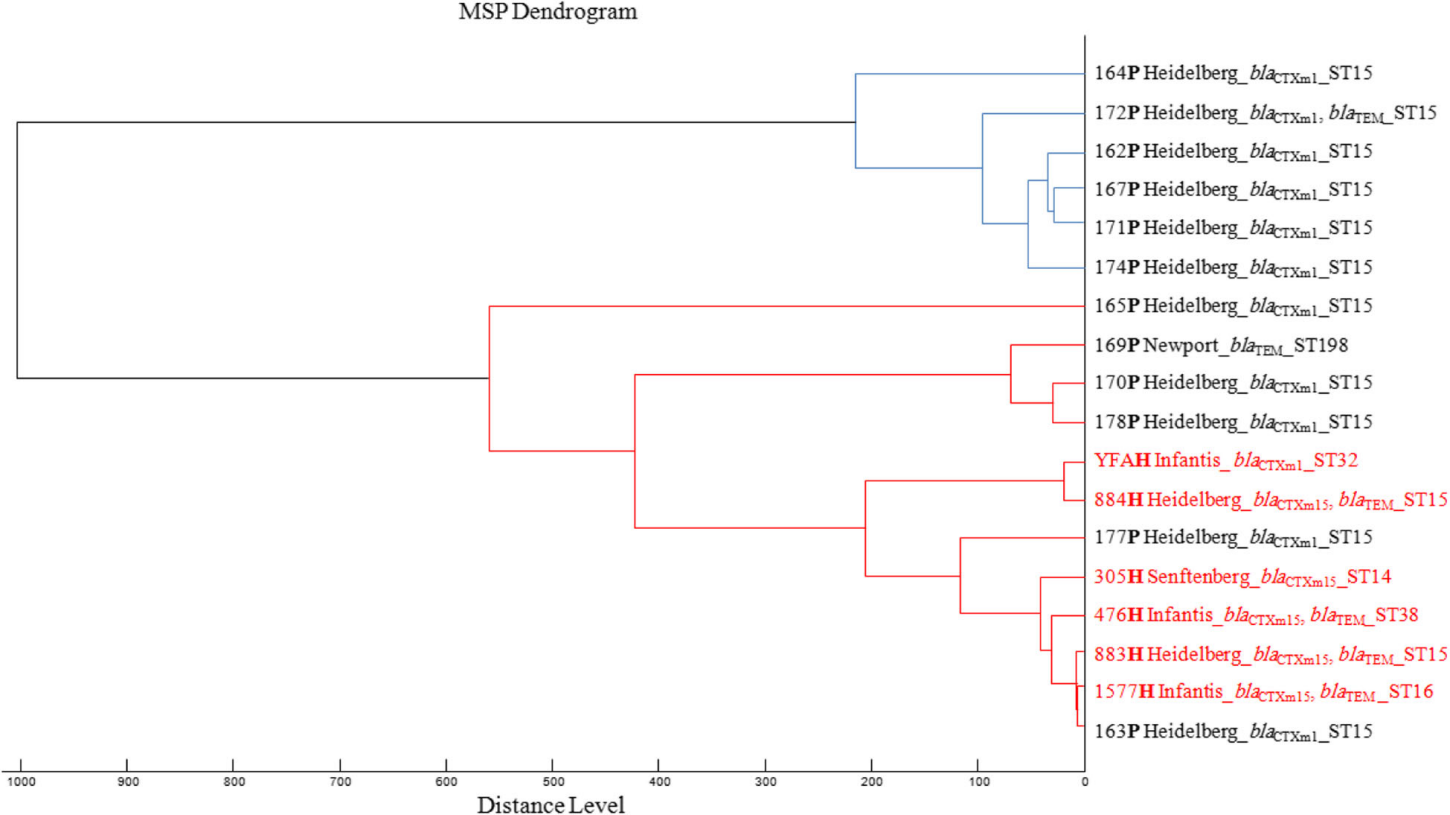
MALDI-TOF MS-based phylogenetic tree of ESBL*-*positive *Salmonella enterica* strains from humans (H) and poultry (P)

The MLST analysis showed that the *salmonella* isolates belonged to six different sequence types (ST) including, ST14, ST15, ST16, ST32, ST38 and ST198 (Fig. 2). The results demonstrate that ST15 represents the predominant clone. Indeed, this ST was found in 13 *Salmonella* ser. Heidelberg strains (two human and 11 avian) (Fig. 2). In addition, the phylogenetic tree shows that seven *Salmonella* ser. Heidelberg strains (two human and five avian) clustered together and belonged to the same sequence type ST15 (Fig. 2), which suggests a possible crossing of this serotype, and particularly this ST between the poultry and the human community in northeastern Algeria.

## Discussion

This study involved thirty-two poultry farms and five slaughter houses. It covered most of the districts in the Skikda province. The selection of the sites was based on the managers’ willingness to cooperate with the study and to spend a significant amount of time and effort to perform and collect the various samples. This work provides epidemiological data on *Salmonella* serotype contamination in poultry farms and slaughterhouses in the region of Skikda, in order to investigate the molecular mechanisms of ß-lactam resistance in avian and human ESBL-producing *S. enterica* and to analyze the genetic relatedness of avian and human isolates.

The recorded prevalence rates (34.37% for poultry farms and 100% for slaughterhouses) are in accordance with those reported in Constantine and Batna provinces (northeastern Algeria): 36.6 and 60%, respectively [29, 30], but were higher than those reported in several European countries (Italy (9.2%), France (3.4%), Germany (2.7%), Spain (1.02%)) and Morocco (24%) [31–33]. This high prevalence can be attributed to the absence of a *Salmonella* infection-control plan (especially in healthy chicken flocks) [31] and to the poor hygienic state in poultry farms, where *Salmonella* can persist during several grow-outs [34]. Furthermore, our findings are in accordance with those of Gardel et al. (2003), who found that samples taken at the third week of the grow-out to be more contaminated by *Salmonella spp.* than those collected at advanced ages [35]. According to many surveys, contamination of poultry products with *Salmonella* may take place at different stages of the production process [36]. After contamination of the birds at the farm, bacteria colonize their intestines and can infect their carcasses at slaughter [37].

Elgroud et al. [29] found that 73.3% of the poultry slaughterhouses in Constantine were *Salmonella*-positive [29]. Among the avian serotypes isolated in our study, Kentucky was the most predominant. Currently, this serotype is distributed worldwide, especially its ST198 [3]. *Salmonella* ser. Heidelberg has been isolated from broiler carcasses collected at four provinces in the center of Algeria [38]. Despite the fact that we have isolated *Salmonella* ser. Enteritidis in only 9% of the samples, it is worth noting that this serotype is the most common in animal products, especially poultry [31]. Our *Salmonella* strains exhibited a high resistance rate to fluoroquinolones, and interestingly, a Kentucky ciprofloxacin-resistant serotype was isolated in France from a patient who had previously stayed in Algeria [39]. In the present study, we report the presence of ESBLs in *Salmonella* ser. Heidelberg*,* Senftenberg*,* Infantis and Newport of avian and human origins, with the CTX-M groups as the most prevalent. To our knowledge, this is the first report of *bla*_CTX-M-1_ genes in avian *Salmonella* strains in Algeria. In fact, this group is the principal ESBL type in human *Salmonella* encountered in Europe [9]. CTX-M-15 was identified in all human *Salmonella* strains. This finding corroborates well with several studies performed in Algeria showing that different *Salmonella* serotypes (Heidelberg, Kedougou, Infantis and Enteritidis) isolated from humans harbored this gene [40–43].

The present study had demonstrated the presence of *bla*_TEM_ genes in one avian *Salmonella* ser. Newport, one avian *Salmonella* ser. Heidelberg strain and all human ESBL-positive strains. Olesen et al. (2004) reported that in Denmark that the major recorded ESBL was the TEM group [44].

In Algeria, cephalosporin use is uncommon in poultry production [22], and the fact that ESBL-positive *Salmonella* strains of avian origin were isolated suggest that these resistant strains may have been introduced into the poultry production chain from other sources, or resulted from the acquisition by avian *Salmonella* strains of ESBL resistance determinants that are generally carried on mobile genetic elements (such as plasmids) [45].

The fact that seven avian and human *Salmonella* ser. Heidelberg strains were of the same ST and clustered together suggests that this clone is circulating in the poultry production chain as well as in the human community. Chicken-to-human transmission of *Salmonella* during farming has been widely demonstrated. It may take place through the food chain [46], and also through occupational exposure from direct contact with live animals and their environment in the broiler chicken industry [47].

## Conclusion

The results of this study demonstrate that *Salmonella* contamination is highly prevalent in broiler poultry farms and slaughterhouses in the region of Skikda (northeastern Algeria), with an increasing resistance to medically important antibiotics. To the best of our knowledge, our results present for the first time, the emergence of ESBL-producing *S. enterica* isolates in poultry in this region. In addition, despite the different sampling times of avian and human *Salmonella* strains, their relatedness has been clearly demonstrated. The clonal relationship between human and avian strains indicates that the poultry industry may act as an important reservoir for ESBL-producing *Salmonella* that are transmitted to humans by direct contact or essentially through the food chain, but more discriminatory typing methods may be able to add more information as to the epidemiology of ESBL-producing *Salmonella* strains in Algeria. Infections caused by multidrug-resistant *Salmonella* species and therapeutic failures increase the risk of death. This is why surveillance programs, rational use of antibiotics and strict biosecurity measures have to be implemented in order to identify the sources, the exact routes of bacterial transmission and to limit the spread of these health-threatening bacteria in the local and national poultry industries.

## Additional files

Additional file1: Main characteristics of patients with the corresponding serotype and *Salmonella* strain ID n°. (DOC 77 kb)

Additional file 2: Results of antibiotics sensitivity testing of poultry and human strains. (XLS 39 kb)

## Abbreviations

DDST: Double-disc synergy test
ESBLs: Extended spectrum β-lactamases
MALDI-TOF: Matrix-assisted laser desorption/ionization time-of-flight.
MLST: Multilocus sequence typing
PCR: Polymerase chain reaction
PWB: Peptone water broth
ST: Sequence type
UTH: University Teaching Hospital
