# Supplementary material for: Prevalence and clonal relationship of ESBL-producing Salmonella strains from humans and poultry in northeastern Algeria
Source: BMC Vet Res. 2017 May 15;13:132. doi: 10.1186/s12917-017-1050-3 (PMC5433073; doi:10.1186/s12917-017-1050-3)
Supplement: Supplementary file 1 — Main characteristics of patients with the corresponding serotype and Salmonella strain ID n°. (DOC 77 kb) [file 12917_2017_1050_MOESM1_ESM.doc]

**Additional File 1.** Main characteristics of patients with the corresponding serotype and *Salmonella* strain ID n°

| **Human strains ID n°** | **Serotypes** | **Origin /Wards** | **Age of patients** | **sex of patients** | **Nature of samples** |
| --- | --- | --- | --- | --- | --- |
| 41 | Typhimirium | Infectious diseases | 40 years | M | Stools |
| 16 | Kentucky | Neonatology | 1 month | M | Stools |
| 1577 | Infantis | Neonatology | 17 days | M | Stools |
| 703 | Kentucky | Neonatology | 10 days | F | Stools |
| 874 | Typhimirium | Infectious diseases | 25 years | M | Stools |
| 2 | Enteritidis | Pediatric | 14 years | F | Stools |
| 753 | Seftenberg | Neonatology | 23 days | F | Stools |
| 884 | Heidelberg | Neonatology | 6 days | F | Stools |
| 953 | Seftenberg | Neonatology | 1 month | M | Stools |
| 854 | Heidelberg | Neonatology | 10 days | M | Stools |
| 669 | Heidelberg | Neonatology | 12 days | M | Stools |
| 864 | Infantis | Neonatology | 15 days | F | Stools |
| 883 | Heidelberg | Neonatology | 17 days | M | Stools |
| 568 | Kedougou | Neonatology | 10 days | F | Stools |
| 1576 | Infantis | Neonatology | 15 days | F | Stools |
| 1623 | Enteritidis | Pediatric | 10 years | F | Stools |
| 3832 | Arizona | Pediatric | 13 years | M | Stools |
| 1664 | Enteritidis | Pediatric | 8 years | M | Stools |
| 587 | Kedougou | Neonatology | 13 days | M | Stools |
| 864 | Infantis | Neonatology | 8 days | M | Stools |
| 524 | Enteritidis | Pediatric | 8 years | F | Stools |
| 256 | Seftenberg | Neonatology | 5 days | F | Stools |
| 209 | Enteritidis | Pediatric | 6 years | M | Stools |
| 483 | Seftenberg | Neonatology | 10 days | F | Stools |
| 1126 | Ohio | Neonatology | 12 days | M | Stools |
| 435 | Kedougou | Neonatology | 7 days | M | Stools |
| 535 | Enteritidis | Infectious diseases | 33 years | F | Stools |
| 1960 | Typhimirium | Infectious diseases | 41 years | F | Blood culture |
| 270 | Kedougou | Neonatology | 10 days | M | Stools |
| 365 | Seftenberg | Neonatology | 5 days | M | Stools |
| 402 | Infantis | Neonatology | 7 days | M | Stools |
| YFA | Infantis | Neonatology | 19 days | F | Stools |
| 604 | Typhimirium | Infectious diseases | 50 years | M | Stools |
| 305 | Seftenberg | Neonatology | 7 days | F | Stools |
| 476 | Infantis | Neonatology | 9 days | M | Stools |
| 343 | Kedougou | Neonatology | 10 days | M | Stools |
| 366 | Infantis | Neonatology | 4 days | F | Stools |

**ID n°**: identification number; **F**: female; **M**: male.
